# Comparison of Compositions and Antimicrobial Activities of Essential Oils from Chemically Stimulated Agarwood, Wild Agarwood and Healthy *Aquilaria sinensis* (Lour.) Gilg Trees 

**DOI:** 10.3390/molecules16064884

**Published:** 2011-06-14

**Authors:** Huaiqiong Chen, Yun Yang, Jian Xue, Jianhe Wei, Zheng Zhang, Hongjiang Chen

**Affiliations:** 1 Key Laboratory of Bioactive Substances and Resources Utilization of Chinese Herbal Medicine, Ministry of Education, Institute of Medicinal Plant Development, Chinese Academy of Medical Sciences & Peking Union Medical College, 100193, Beijing, China; Email: chenhuaiqiong@implad.ac.cn (H.C); jxue@implad.ac.cn (J.X); zhangzheng@implad.ac.cn (Z.Z.); 2 Hainan Branch Institute of Medicinal Plant (Hainan Provincial Key Laboratory of Resources Conservation and Development of Southern Medicine), Chinese Academy of Medical Sciences & Peking Union Medical College, 571533, Wanning, China; Email: yangyun43@gmail.com (Y.Y.); hjchen@implad.ac.cn (H.C.)

**Keywords:** *Aquilaria sinensis* (Lour.) Gilg, healthy trees, agarwood, essential oil, GC-MS, antimicrobial activity

## Abstract

The composition and antimicrobial activity of the essential oils which were obtained from agarwood originated from *Aquilaria sinensis* (Lour.) Gilg stimulated by the chemical method (S1) were characterized, taking wild agarwood (S2) and healthy trees (S3) respectively as the positive and negative controls. The chemical composition of S1 was investigated by gas chromatography-mass spectrometry (GC-MS). The essential oil of S1 showed a similar composition to that of S2, being rich in sesquiterpenes and aromatic constituents. However, the essential oil of S3 was abundant in fatty acids and alkanes. Essential oils of S1 and S2 had better inhibition activities towards *Bacillus subtilis* and *Staphyloccus aureus*, compared with essential oil of S3. *Escherichia coli* was not sensitive to any of them.

## 1. Introduction

Agarwood, a highly valuable resinous and fragrant heartwood, is used as incense for religious ceremonies, perfumes in the Arab world, ornamental materials, and medicinal components in oriental medicine [1,2]. It comes from the damage caused to healthy trunks or branches of the trees of some *Aquilaria* species in the family Thymelaeaceae by mold. As a healthy tree *Aquilaria* is worth next to nothing, but high-quality agarwood can fetch as much as US$1,000 per kilogram [3]. In a natural environment, it often takes several years for a wild damaged *Aquilaria* species plant to form agarwood [3]. The over-use of agarwood has seriously affected the natural resources of all *Aquilaria* species capable of producing agarwood, thus making these endangered species listed in Appendix II of the Convention on Internal Trade in Endangered Species of Wild Fauna and Flora (CITES) since 2004 [4]. 

In order to meet the demand for agarwood and protect the wild *Aquilaria* trees, many countries have been developing *Aquilaria* plantationsf [5,6,7]. *A.* *sinensis*, the main plant resource in China for agarwood, is chiefly distributed in South China [8]. *A. sinensis* trees are now widely cultivated in Hainan and Guangzhou provinces, with the planting area estimated to cover more than 700 acres. 

The main active compounds in agarwood have been revealed to be sesquiterpenes and 2-(2-phenylethyl) chromone derivatives [9]. In order to improve the planting value of *Aquilaria* trees, great efforts have been made to induce healthy trees to produce these sesquiterpenes and 2-(2-phenylethyl) chromone derivatives, consequently forming argarwood [5,10]. The common methods now used in China and other countries include the deliberate wounding of trees with large knives and the hammering of nails into tree trunks. A chemical treatment method has also been developed recently [11]. Meanwhile, some studies have been carried out to compare the quality of man-treated and wild agarwood. Tamuli and Bhuiyan studied the quality of agarwood (*A.* *agallocha* Roxb.) formed through fungal infection by GC-MS [12,13]. Dai Haofu *et al.* evaluated the quality of three Chinese agarwood (*A.* *sinensis*) samples produced by the methods of nail insetting, holing and trunk breaking, respectively, through GC-MS [14], but there are no reports about agarwood formed by chemical methods. In this study, in order to test the quality of the agarwood originated from *A. sinensis* stimulated by the chemical method (S1), its chemical composition and relative amount of essential oils were measured by GC-MS, taking the wild agarwood (S2) and healthy trees (S3) as controls. The antimicrobial activities of essential oils of the agarwood originating from *A.* *sinensis* were also determined.

## 2. Results and Discussion

### 2.1. Chemical Composition of the Essential Oils

**Figure 1:**
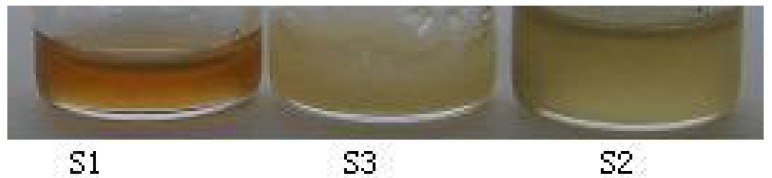
Color and state of the essential oils of three tested samples at room temperature.

The yields of essential oils obtained after hydrodistillation of S1, S2 and S3 were 0.042% (*w*/*w*), 0.32% (*w*/*w*) and 0.0128% (*w*/*w*), respectively. They showed different colors and states (Figure 1). At room temperature, the essential oil of S1 was yellow, aromatic and a liquid, similar to that of S2, but different from that of S3. The essential oil of S2 was green, aromatic and liquid. The essential oil of S3 had an acidic smell and was a solid at room temperature.

**Figure 2:**
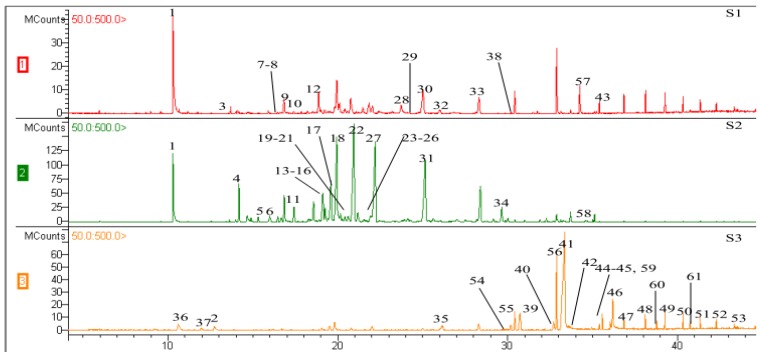
GC chromatograms of the three essential oils. Component numbers in the chromatogram come from Table 1.

A total of sixty-one essential compounds were identified from the three samples (Table 1 and Figure 2). Forty-two components were identified in S1, representing 90.01% of the total volatiles, with the major constituents being sesquiterpenes and aromatics, such as guaia-1(10),11-dien-9-one (10.89%), guaiol (9.34%), benzylacetone (7.91%) and hinesol (6.34%). Thirty-six components were identified in S2, representing 92.16% of the total volatiles. The dominant compounds were baimuxinal (14.78%), guaiol (10.67%), α-copaen-11-ol (10.22%) and 1,2,5,5,8a-pentamethyl-1,2,3,5,6,7,8,8a-octahydro-naphthalen-1-ol (5.82%). Thirty components were identified in the essential oil of S3, representing 95.68% of the total volatiles. *N*-hexadecanoic acid and oleic acid accounted for 59.65% of the total essential oil, which explained its smell and state. 

The investigation showed that the essential oil of S1 had similar components to those of S2. They were both rich in sesquiterpenes and aromatics, which reached 80.00% in S1 and 89.01% in S2. Thirty-one common compounds were identified in S1 and S2. Benzylacetone, cubenol, guaiol, eudesm-7(11)-en-4α-ol, α-copaen-11-ol, and baimuxinal were found to be the six compounds (in bold in Table 1) that had high relative amounts both in S1 and S2. They might be used as the reference compounds in determining the quality of agarwood. 

The essential oil of S1 and S2 had significantly different components from that of S3, which had abundant fatty acid and alkanes. For instance, a trace of *n*-hexadecanoic acid was found in the oils of S1 and S2, whereas it reached 49.47% in S3; oleic acid was 10.18% in S3, but it was totally absent in S1 and S2. As to the alkanes, S1 and S3 were very similar, and most of the alkanes identified in S3 were also identified in S1, whereas no alkanes were detected in S2. This may affect the complex process of agar accumulation and the prolonged duration of accumulation, as well as the contents of fatty acid and alkanes [13]. A higher oil yield would require a longer time for resin formation.

It has been reported that different artificial methods used to stimulate agarwood formation in *Aquilaria* result in different agarwood qualities. Tamuli and Bhuiyan both showed that the essential oils obtained from the plants (*A. agallocha* Roxb.) inoculated with fungus, *i.e.*, *Chaetomium* globosum, for 30 days or from plants injected artificial screws showed similar component distributions with that of healthy trees according to GC-MS [12,13]. Dai *et al.* found that the essential oils of the agarwood produced by nail insetting and holing for two years were full of sesquiterpenes and aromatic constituents, while the essential oil of agarwood formed through trunk breaking for two years was full of fatty acids [14]. Comparing our results with the above results, we come to the conclusion that the chemical stimulation method is a simple and efficient way of inducing *Aqularia* plants to form resinous material.

As to the total of 8.93% sesquiterpene compounds identified in essential oil of healthy trees (S3), it was probably because the cutting process was sufficient damage to initiate the resinous material formation process. During the drying period at room temperature, cells might be still alive, and the sesquiterpene metabolic pathways were thus initiated.

**Table 1:** Chemical compositions and relative amounts of the essential oils from S1, S2 and S3.

No.		Compounds	RI a	Relative amount (%) c			Identification
				S1	S2	S3	
	**Sesquiterpenes and aromatics**			80.00	89.01	8.93	
**1**		Benzylacetone *	**1257**	**7.91**	**2.34**	- ^b^	RI,MS,
**2**		Vanillin	1418	-	-	0.46	RI[15],MS
**3**		*α*-Humulene	1464	0.30	-	-	RI,MS,[16,17]
**4**		*α*-Selinene	1493	0.45	3.71	-	RI[18],MS
**5**		*α*-Agarofuran	1535	-	0.24	-	RI[18],MS
**6**		Elemol	1556	0.32	0.71	-	RI[19,24],MS
**7**		2,6-Dimethyl-10-methylene-12-oxatricyclo[7.3.1.0(1,6)]tridec-2-ene	1576	0.37	0.61	-	MS
**8**		5*β*,7*β*H,10*α*-Eudesm-11-en-1*α*-ol	1583	0.54	0.66	-	MS
**9**		Caryophyllene oxide	1588	2.22	2.12	-	RI[19,24],MS
**10**		2H-Benzocyclohepten-2-one, 3,4,4a,5,6,7,8,9-octahydro-4a-methyl-, (S)-	1593	-	0.20	-	MS
**11**		Isoaromadendrene epoxide	1606	0.94	2.77	-	RI[20],MS
**12**		*γ*-Eudesmol	**1632**	**1.06**	**2.84**	0.50	RI[21],MS[22]
**13**		Hinesol	1638	6.34	0.34	-	RI[23],MS
**14**		Agarospirol	1643	0.80	4.03	0.85	RI[24],MS
**15**		**Cubenol**	**1647**	**2.21**	**1.97**	-	RI[25],MS
**16**		*cis*-Z-*α*-Bisabolene epoxide	1651	0.83	0.78	-	RI[26],MS
**17**		(-)-Aristolene	1654	0.61	4.70	1.31	MS[22]
**18**		**Guaiol**	**1661**	**9.34**	**10.67**	2.18	MS[27,28,29]
**19**		**Eudesm-7(11)-en-4*α*-ol**	**1666**	**4.35**	**2.09**	-	RI,MS[30]
**20**		Aromadendrene oxide (1)	1674	1.27	1.41	-	RI[31],MS
**21**		6-Isopropenyl-4,8a-dimethyl-1,2,3,5,6,7,8,8a-octahydro-naphthalen-2-ol	1678	1.19	1.33	-	RI[20],MS
**22**		***α*** **-Copaen-11-ol**	**1686**	**4.06**	**10.22**	-	RI,MS[32]
**23**		4,4,11,11-tetramethyl-7-tetracyclo-[6.2.1.0(3.8)0(3.9)]undecanol	1690	0.53	1.50	-	MS
**24**		Bicyclo[4,4,0]dec-2-ene-4-ol,2-methyl-9-[prop-1-en-3-ol-2-yl]	1697	3.11	0.52	-	MS
**25**		Diepi-α-cedrene epoxide	1701	6.00	0.38	-	MS
**26**		Aromadendrene oxide (2)	1705	-	1.88	-	RI[33],MS
**27**		**Baimuxinal**	**1707**	**2.44**	**14.78**	1.52	MS[27,28,32]
**28**		Selina-3,11-dien-14-al	1733	5.50	0.38	-	RI[18], MS
**29**		5(1H)-Azulenone, 2,4,6,7,8,8a-hexahydro-3,8-dimethyl-4-(1-methylethylidene)-, (8*S*-cis)-	1736	0.21	1.03	-	RI,MS
**30**		Guaia-1(10),11-dien-9-one	1753	10.89	-	-	RI[18],MS
**31**		1,2,5,5,8a-Pentamethyl-1,2,3,5,6,7,8,8a-octahydronaphthalen-1-ol	1755	-	5.82	-	RI[34],MS
**32**		6-Isopropenyl-4,8a-dimethyl-3,5,6,7,8,8a-hexahydro-2(1H)-naphthalenone	1769	1.65	0.54	-	RI[34],MS
**33**		Eremophila-7(11),9-dien-8-one	1811	4.54	5.42	2.11	RI[34],MS
**34**		Acetic acid, 3-hydroxy-6-isopropenyl-4,8a,dimethyl-1,2,3,5,6,7,8,8a-octahydronaphthalen-2-yl ester	1847	-	3.04	-	RI[34],MS
	**Fatty acid and Alkanes**			5.75	0.64	79.31	
**35**		Tetradecanoic acid	1772	-	-	2.36	RI[35],MS
**36**		Nonanoic acid	1278	-	-	1.50	RI[36],MS
**37**		*n*-Decanoic acid	1371	-	-	0.52	RI[36],MS
**38**		*cis*-5-Dodecenoic acid	1863	0.20	-	-	RI[34],MS
**39**		Pentadecanoic acid	1878	-	-	4.87	RI[34],MS
**40**		*cis*-9-Hexadecenoic acid	1955	-	-	2.87	RI[34],MS
**41**		*n*-Hexadecanoic acid	1982	0.30	0.06	49.47	RI[34],MS
**42**		Hexadecanoic acid, ethyl ester	1996	-	-	1.13	RI[34],MS
**43**		Eicosane	1999	0.22	0.58	-	RI[34],MS
**44**		Heptadecanoic acid	2073	-	-	0.37	RI[34],MS
**45**		Heneicosane	2100	0.51	-	1.09	RI[34],MS
**46**		Oleic Acid	2153	-	-	10.18	RI[34],MS
**47**		Docosane	2200	0.80	-	0.53	RI[34],MS
**48**		Tricosane	2300	0.97	-	0.80	RI[34],MS
**49**		Tetracosane	2400	0.79	-	0.86	RI[34],MS
**50**		Pentacosane	2500	0.70	-	0.87	RI[34],MS
**51**		Hexacosane	2600	0.62	-	0.80	RI[34],MS
**52**		Heptacosane	2700	0.45	-	0.57	RI[34],MS
**53**		Octacosane	2800	0.20	-	0.54	RI[34],MS
	**Others**			4.26	2.51	7.44	
**54**		2-Hydroxycyclopentadecanone	1851	0.24	0.30	2.32	RI[37],MS
**55**		1,2-Benzenedicarboxylic acid, bis(2-methylpropyl) ester	1869	0.69	-	0.85	RI[37],MS
**56**		Dibutyl phthalate	1962	2.13	1.23	2.47	RI[37],MS
**57**		1,2,3,4-Tetrahydro-1-nonylnaphthalene	2021	0.64	-	-	MS
**58**		8,9-Dehydro-9-formyl-cycloisolongifolene	2082	0.56	0.98	-	MS
**59**		*γ*-Palmitolactone	2111	-	-	0.99	RI[34],MS
**60**		4,8,12,16-Tetramethylheptadecan-4-olide	2357	-	-	0.42	RI[34],MS
**61**		1,2-Benzenedicarboxylic acid, mono(2-ethylhexyl) ester	2541	-	-	0.39	RI[37],MS
	**TOTAL**			90.01	92.16	95.68	

Compounds are listed in the order of elution; ^a^ RI indicates the retention indices which were calculated against C_8_-C_40_ *n*-alkanes on the non-polar VF-5MS column; ^b^ not detected; ^c^ Relative amount indicates the relative amount (the peak area relative to the total peak area); * verified by the authentic compound.

### 2.2. Antimicrobial Activities

Table 2 shows the antimicrobial activities of the three essential oils. The results indicated that all of the three essential oils had some activity against almost all the tested bacteria. All the essential oils were more active against Gram-positive bacterial strains (*S. aureus* and *B. subtilis*) than against the Gram-negative bacterial strain *E. coli*. Taken together, especially the results of MICs and MBCs, the essential oils of S1 and S2 had better antimicrobial activities than that of S3. This is probably because the essential oils of S1 and S2 were full of sesquiterpenes and aromatics, which may include some active components. It’s known that sesquiterpenes usually possess antimicrobial activity. It is critical to note that, if active components are isolated and purified, their antimicrobial activities could become stronger. 

**Table 2:** Screening results for antimicrobial activity of the three essential oils.

Essential oil		*E. coli*	*S. aureus*	*B. subtilis*
**S1**	AWD ^a^	8.14 ± 0.05	10.29 ± 0.20	11.68 ± 0.26
	MIC ^b^	6.25	3.125	0.39
	MBC ^c^	25	6.25	12.5
**S2**	AWD ^a^	7.00 ± 0.02	11.25 ± 0.02	11.57 ± 0.02
	MIC ^b^	6.25	0.195	0.195
	MBC ^c^	12.5	6.25	12.5
**S3**	AWD ^a^	7.52 ± 0.26	8.10 ± 0.24	9.39 ± 0.30
	MIC ^b^	12.5	1.56	0.78
	MBC ^c^	>25	12.5	>25
**Gentamicin**	AWD ^a^	23.08 ± 0.88	21.45 ± 1.77	23.73 ± 0.32
	MIC ^b^	0.487	0.487	0.487
	MBC ^c^	0.487	0.487	0.487
**DMSO**	AWD ^a^	0	0	0
**ddH_2_O**	AWD ^a^	0	0	0

^a^ AWD = agar well diffusion method. The diameters of the inhibition zone, including the well diameters, are 6 mm; ^b^ MIC = minimum inhibitory concentration. The values of the three oil samples are given in mg/mL, and the values of gentamicin are given in μg/mLp; ^c^ MBC = minimum bactericidal concentration. The values of the three oil samples are given in mg/ml, and the values of Gentamicin are given in μg/mL.

The negative-control experiments, including the antimicrobial test for DMSO and ddH_2_O, indicated no microbial contamination in the essential oils and media, as shown by the data in Table 2. 5% *v*/*v* DMSO, the maximum concentration used for dissolving essential oil, showed no inhibition on the microbial growth.

This is the first report concerning the antimicrobial activities to the three bacterial strains of Chinese agarwood oil from *A.* *sinensis*. In previous studies, Mei [28] showed that the essential oil from Chinese agarwood had anti-MRSA activity; Wetwitayaklung [18] found that the essential oil of agarwood (*A.* *crassna*) had antimicrobial activities against *S.* *aureus* and *C.* *albicans*, but it was not active against *E. coli* at the maximum study concentration (2 mg/mL). In this study, the MICs and MBCs to *E. coli* were more than 6.25 and 12.5 mg/mL, respectively, consistent with the report by Wetwitayaklung.

## 3. Experimental

### 3.1. Plant Material

The three types of samples included artificially chemically stimulated plants, wild agarwood and stems of six-year-old healthy trees (Table 3). *A.* *sinensis* trees were cultivated wild in Haikou City, Hainan Province. The chemical treatment method was used to stimulate the formation of resinous in *A. sinensis* trees. A chemical reagent (NaCl) of a certain concentration was injected slowly into the xylem part of a tree. Because of water transport, the chemical reagent was transported to the whole body of the tree, thus forming an overall injury in the tree. This would stimulate the whole tree to produce resinous material to defend from the damage. Healthy trees without any treatment were collected as the negative control. The wild agarwood was purchased from Guangxi Yulin Market and identified by Dr. Jianhe Wei. 

**Table 3:** Materials used in this study.

Brief Name	Stimulating method	Characterization	Age	Plant origin
S1	chemical method	agarwood	6 years	*A. sinensis*
S2	unknown natural factor	agarwood	unknown	*A. sinensis*
S3	no damage	healthy trees	6 years	*A. sinensis*

### 3.2. Isolation of Essential Oils

Three samples were powdered, passed through 20 mesh sieves, soaked in water overnight and then subjected to hydrodistillation for 12 h using a Clevenger apparatus. The distilled oil was dried over anhydrous sodium sulfate and stored in a freezer at −20 °C until analysis.

### 3.3. GC-MS Analysis

GC-MS analysis was performed with a Varian 450 gas chromatograph (USA) equipped with a VF-5MS capillary column (30 m × 0.25 mm i.d., flim thickness 0.25 μm) and a Varian 300 mass spectrometer with an ion trap detector in full scan mode under election impact ionization (70 eV). The carrier gas was helium, at a flow rate of 1 mL/min. The injections were performed in splitless mode at 250 °C. 1 μL essential oil solution in hexane (HPLC grade) was injected. The operating parameters were the temperature program of 50 °C for 1 min, ramp of 10 °C/min up to 155 °C (15 min), subsequent increase to 280 °C with an 8 °C/min heating ramp, and keeping at 280 °C for 10 min. The scan range was 50–500 amu under full scan. 1 μL C_8_-C_40_ n-alkanes was injected separately and ran in the same program as the essential oils. 

### 3.4. Identification of Components

Most constituents were identified via gas chromatography by comparing their Kovats retention indices (RI) with those from the literature, and computer matching against the NIST 08 and NIST Chemistry WebBook (http://webbook.nist.gov/chemistry/) databases. The Kovats retention indices were determined in relation to a homologous series of *n*-alkanes (C_8_–C_40_) under the same operating conditions. AMDIS software was used to calculate Kovats retention indices. Further identification was made by comparing their mass spectra with these stored in NIST 08 and with mass spectra from the literature [15,16,17,18,19,20,21,22,23,24,25,26,27,28,29,30,31,32,33,34,35,36,37]. The relative concentrations of the components were obtained by peak areas normalization without applying correction factors.

### 3.5. Antimicrobial Activity

#### 3.5.1. Test Microorganisms

Three clinical bacteria, *Staphylococcus aureus* ATCC 25923, *Bacillus subtilis* ACCC11060 and *Escherichia coli* ATCC25922, were used as test organisms in the screening. The microbial strains were obtained from the College of Life Science, Capital Normal University, Beijing, China.

#### 3.5.2. Determination of Diameters of Inhibition Zone

Simple susceptibility screening test through agar well diffusion method was used [28]. The inocula of the bacterial strains were adjusted to 0.5 McFarland standard turbidity (approximately 10^8^ CFU/mL) [38,39]. One hundred μL of a suspension containing approximately 10^8^ CFU/mL of each microorganism was spread on nutrient agar (NA). Six-millimeter diameter wells were cut from the agar using a sterile cork-borer, and 50 μL of the oil solution in a concentration of 50 mg/mL (dissolved in DMSO) were delivered into the wells. Negative controls were prepared using DMSO. Gentamincin (100 μg/mL) were used as the positive reference standards. The plates were incubated for 18–24 h at 37°C. The antimicrobial activity was evaluated by measuring the zone of inhibition against the test organisms.

#### 3.5.3. Determination of Minimum Inhibitory Concentration (MIC)

The MICs of the samples against the test bacterial strains were determined by the micro-well dilution method. The inocula of the bacterial strains were adjusted to 0.5 McFarland standard turbidity (approximately 10^8^ CFU/mL). The essential oils were first dissolved in 10% DMSO, and serial two-fold dilutions of the oil were prepared in a 96-well plate, ranging from 3.9 mg/mL to 50 mg/mL. The MIC was defined as the lowest concentration of the essential oil at which the microorganism does not demonstrate visible growth [38,39,40]. Microorganism growth was indicated by turbidity. The MICs of the standard (gentamicin) were also determined in the same experiments.

#### 3.5.4. Determination of Minimum Bactericidal Concentration (MBC)

Referring to the results of the MIC assays, the wells showing complete absence of growth were identified and 10 μL of each well was transferred to agar plates and incubated at 37 °C for 24 h. The MBC was defined as the lowest concentration of the juniper essential oil that allows no growth of microorganisms [40,41,42]. The MBCs of the standard (gentamicin) were also determined in the same experiments.

#### 3.5.5. Data Analysis

All experiments were repeated at least twice. The data were recorded as means±standards and were analyzed with SPSS (version 13.0 for windows, SPSS Inc.). 

## 4. Conclusions

The characterization of the essential oil obtained from the agarwood originated from *A. sinensis* stimulated by the chemical method has very high similarity with that of the essential oil of wild agarwood, both in chemical composition and antimicrobial activity. This suggests that agarwood could be produced by the artificially chemically stimulation method. What chemical agents and duration would be suitable for inducing better agarwood formation need further studies. This is the first report concerning the analysis of essential oils from chemically stimulated agarwood.
